# Towards Model-Free Tool Dynamic Identification and Calibration Using Multi-Layer Neural Network

**DOI:** 10.3390/s19173636

**Published:** 2019-08-21

**Authors:** Hang Su, Wen Qi, Yingbai Hu, Juan Sandoval, Longbin Zhang, Yunus Schmirander, Guang Chen, Andrea Aliverti, Alois Knoll, Giancarlo Ferrigno, Elena De Momi

**Affiliations:** 1 Dipartimento di Elettronica, Informazione e Bioingegneria, Politecnico di Milano, 20133 Milano, Italy; 2 Department of Informatics, Technical University of Munich, 85748 Munich, Germany; 3 Department of GMSC, Pprime Institute, CNRS, ENSMA, University of Poitiers, UPR 3346 Poitiers, France; 4 BioMEx Center & KTH Mechanics, KTH Royal Institute of Technology, SE-100 44 Stockholm, Sweden; 5 College of Automotive Engineering, Tongji University, Shanghai 201804, China

**Keywords:** multi-layer neural network, model-free, calibration, tool dynamic identification

## Abstract

In robot control with physical interaction, like robot-assisted surgery and bilateral teleoperation, the availability of reliable interaction force information has proved to be capable of increasing the control precision and of dealing with the surrounding complex environments. Usually, force sensors are mounted between the end effector of the robot manipulator and the tool for measuring the interaction forces on the tooltip. In this case, the force acquired from the force sensor includes not only the interaction force but also the gravity force of the tool. Hence the tool dynamic identification is required for accurate dynamic simulation and model-based control. Although model-based techniques have already been widely used in traditional robotic arms control, their accuracy is limited due to the lack of specific dynamic models. This work proposes a model-free technique for dynamic identification using multi-layer neural networks (MNN). It utilizes two types of MNN architectures based on both feed-forward networks (FF-MNN) and cascade-forward networks (CF-MNN) to model the tool dynamics. Compared with the model-based technique, i.e., curve fitting (CF), the accuracy of the tool identification is improved. After the identification and calibration, a further demonstration of bilateral teleoperation is presented using a serial robot (LWR4+, KUKA, Germany) and a haptic manipulator (SIGMA 7, Force Dimension, Switzerland). Results demonstrate the promising performance of the model-free tool identification technique using MNN, improving the results provided by model-based methods.

## 1. Introduction

Robot control with physical interaction has been widespread and draws a lot of research interests in the past decades [1]. The availability of reliable interaction force information proved to be capable of increasing the control precision and of dealing with the surrounding complex environments, for example, in the context of robot-assisted surgery and bilateral teleoperation [2]. In practical applications, since the force sensor is used for measuring the interaction forces on the tooltip, the force sensor is usually mounted between the end-effector of the robot and its tool. However, the force acquired from the force sensor includes not only the interaction forces but also the gravity force of the tool. Hence the tool dynamic identification is required for accurate dynamic simulation and model-based control. Especially for bilateral teleoperation control, which provides haptic feedback for the surgeon in robot-assisted minimally invasive surgery (RA-MIS) [3,4], achieving accurate force sensing can ease the tasks performing [5] and improve the quality of the surgery, for example, enhancing surgical accuracy [6], optimizing dexterity and minimizing the trauma of the patient [7,8].

To achieve accurate force sensing, several studies have been performed regarding the dynamic model identification of the tool gravity using model-based techniques, like curve fitting (CF). However, it is difficult to identify an accurate mathematical model due to manufacturing and assembly variances. Few of the identified models can be used directly on other robot applications because of the variances of the tool mechanics. Hence, the model-free based tool identification which can be directly applied regardless of the tool mechanics, is suitable. Furthermore, force sensor calibration methodologies must be precise and time compelling in a practical application [9]. The least-squares optimization method has been widely applied for identification and calibration of multi-axis force sensors [10] as one of the traditional calibration methods. However, because it requires a large number of experimental data and ignores the nonlinear characteristics of the robot system, the implementation of this method is hard and not practical [11]. A calibration method using a pre-calibrated force plate was introduced in [12]. Although this method makes the calibration method easier dismantling the sensor remains an issue [13]. A challenging problem with most of the calibration methods [10] proposed in the literature is that they ignored the influence of gravity on the surgical tool and the nonlinear disturbances due to the setup of the tool, which affects the force sensing accuracy in the teleoperation system.

During the past decades, machine learning (ML) techniques have proven to be a powerful tool for regression analysis. As one of the most popular ML algorithms [14,15], artificial neural networks (ANN) have been widely applied to solve various regression problems, such as motion tracking [16,17,18], biomedical applications [19], and geophysical explorations [20]. Most of the adopted single hidden layer (SHL) networks regression methods are proven to obtain a higher accuracy than traditional approaches for nonlinear regression [21,22]. In our previous work, CF and ANN are known for the capacity of modeling the nonlinear mapping relation between multiple inputs and outputs [23]. The performance of CF and ANN are compared in terms of modeling accuracy. However, the prediction error can be reduced to map the multi-inputs to the multi-outputs. In addition, over-fitting and under-fitting are problems of SHL networks for predicting the time-varying curves in a dynamic environment. Adding hidden layers is one aspect of enhancing the accuracy and stability of the regression model. So, multilayer neural networks (MNN) become a popular method applied to the multi-inputs multi-outputs (MIMO) system [24].

This paper utilizes two different MNN structures for tool gravity identification based on feed-forward networks (FF-MNN) and cascade-forward networks (CF-MNN) to model gravity force due to tool’s weight in accordance with the tool direction in the Cartesian space. The two MNN structures are adopted to build a model allowing to improve the performance of nonlinear regression analysis. This model is established based on the collected training dataset, and it is used to map the three-dimensional gravity force ($\left[F_{x},F_{y},F_{z}\right]$) to the 3-D Euler angles of the end effector, namely $\left[\theta_{x},\theta_{y},\theta_{z}\right]$. The performance of the two types of MNN models is compared in terms of modeling accuracy [25], prediction speed and training time.

After the tool dynamics identification, the calibration procedure is achieved with the classic singular value decomposition (SVD) algorithm. The calibration results with different gravity compensation strategies are investigated. Finally, a bilateral teleoperation demonstration is performed to show the transparency of the bilateral teleoperation system when including the proposed model-free tool dynamic identification method.

The following parts of this paper are organized as: Section 2 shows the kinematic model of the serial robot. Section 3 presents the corresponding methodologies, separately. The experiment validation and results of the proposed methodology evaluated with the KUKA LWR4+ robot are described in Section 4. Finally, Section 5 draws a conclusion and delineates avenues for further work.

## 2. Kinematic Model of the Serial Robot

**Figure 1:**
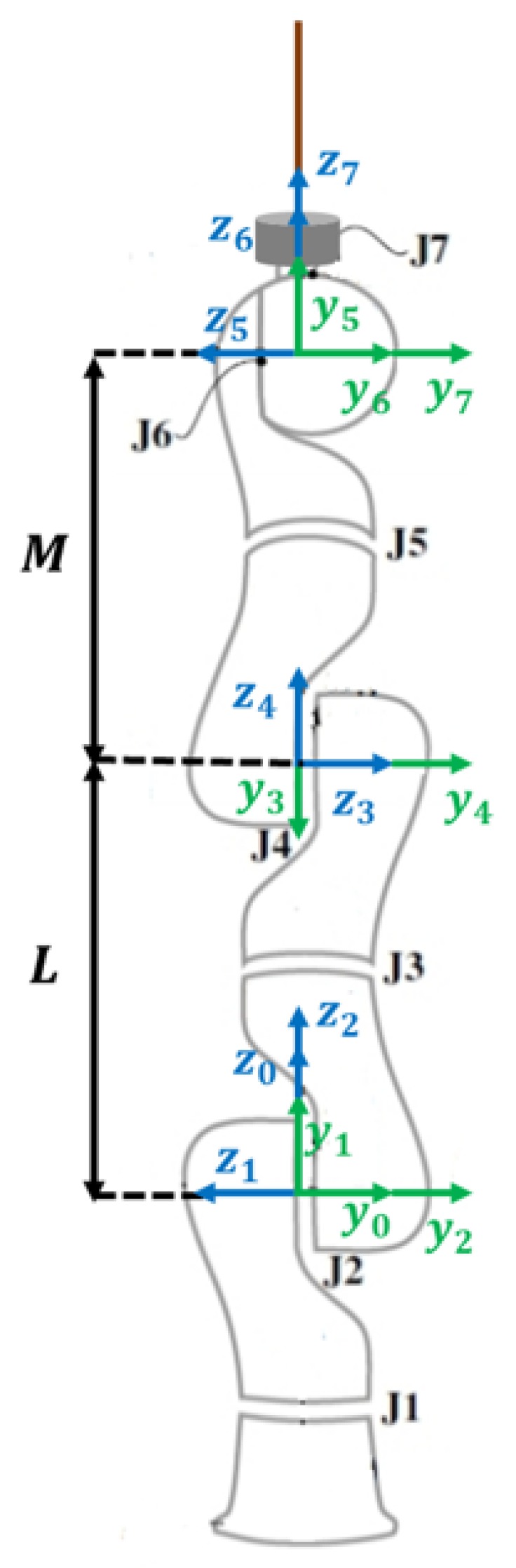
Kinematic structure of KUKA LWR4+ robot. The robot is in its home position [0,0,0,0,0,0,0] with a force sensor mounted between the end effector and the surgical tool.

The kinematic model of an anthropomorphic seven degrees-of-freedom (DoFs) robotic arm (LWR4+, KUKA, Germany) is shown in Figure 1. The corresponding Denavit–Hartenberg (D-H) parameters [26] are listed in Table 1 [27]. Based on the D-H parameters, a homogeneous transformation matrix of two consecutive link frames of the serial robot arm, $\left\{i-1\right\}$ to $\left\{i\right\}$ can be defined as follows [28]:

(1) $$\begin{array}{cc} {}_{i-1}^{i}T= & Rot\left(x_{i-1},\alpha_{i-1}\right)\mathrm{Tr}\left(x_{i-1},a_{i-1}\right)\mathrm{Tr}\left(z_{i},d_{i}\right)\\ & \mathrm{Rot}\left(z_{i},\theta_{i}\right) \end{array}$$

where the transformation matrix ${}_{i-1}^{i}T$ is a composition of rotations and translations to move a frame coincident to frame $\left\{i-1\right\}$ until it coincides with frame $\left\{i\right\}$ [29]. Parameters of link i−1 include the link twist angle $\alpha_{i-1}$, link length $a_{i-1}$, and link offset $d_{i}$, whereas parameters of link *i* include joint variable $\theta_{i}$. Rot and Tr are (4×4) matrices of rotational and translational transformations along an axis, respectively [28]. Therefore, the rotation angle can be obtained from its forward kinematic function.

With the D-H matrix at hand, the transformation matrix from the robot base frame to its end effector frame can be computed using joint angles. The robot tool pose can be obtained by multiplying the link transformation matrix as follows [28]:

(2) $${}_{E}^{0}T{=}_{1}^{0}T_{2}^{1}T_{3}^{2}T_{4}^{3}T_{5}^{4}T_{6}^{5}T_{E}^{6}T$$

where ${}_{i+1}^{i}$*T* is the transformation matrix as shown in (1).

## 3. Methodology

**Figure 2:**
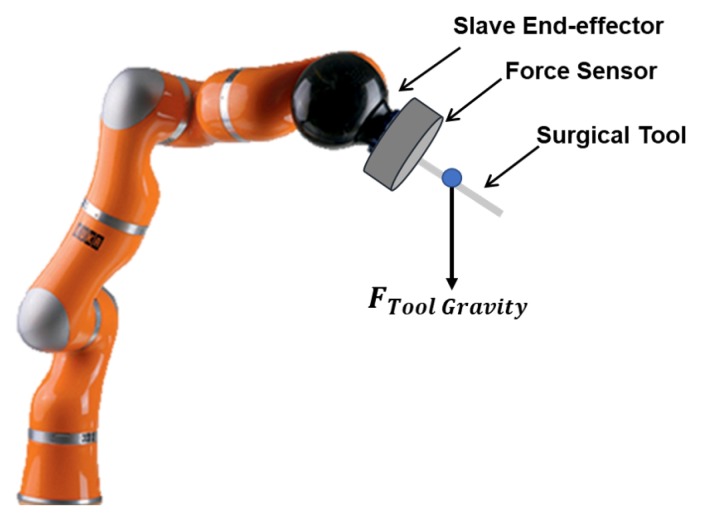
Force sensor installation. It is mounted at the end effector of the manipulator and a robot tool is attached on the force sensor.

As shown in Figure 2, in order to transmit the interaction force on the tooltip to the robot, the force sensor is mounted between the end effector and the tool. The interaction force between the tooltip and the environment is measured. It is obvious that the output of the force sensor includes not only the interaction force but also force generated by load of the tool gravity. Hence, it is essential to develop techniques for tool dynamic identification to eliminate the disturbance from the tool weight and transmit the accurate interaction force to the robot control system.

### 3.1. Tool Dynamics Identification

Due to the influence of the gravitational force of the robot tool, the output of force sensor $F_{S}\in R^{3}$ comprises the gravitational force $F_{ToolGravity}\in R^{3}$ and the interaction force $F_{Interaction}\in R^{3}$ between the tool and the environment. The formula can be written as:

(3) $$F_{S}=F_{ToolGravity}+F_{Interaction}$$

**Figure 3:**
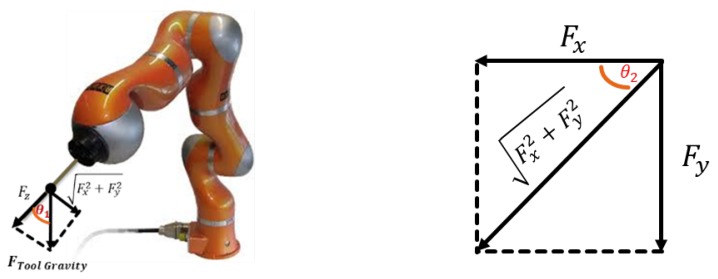
Tool gravity mapped on the force sensor.

With the motion of the robot, the components of the gravity force $F_{ToolGravity}$ on $F_{S}$ vary according to the pose of the robot tool. As shown in Figure 3, the force exerted on the force sensor varies with the tool directions $\theta_{1}$ and $\theta_{2}$. Hence, the output of the force sensor is perturbed and it cannot represent the interaction force accurately. It is essential to identify the tool dynamics and eliminate the influence of the gravity for accurate force sensing.

#### 3.1.1. Model-Based Tool Gravity Identification Using Curve Fitting

According to Figure 3, the orientation matrix of the tool changes with the robot arm placement, and the impact of the weight on the force sensor measurements also varies with $\theta_{1}$ and $\theta_{2}$. Thus, it is essential to consider the gravity identification depending on the orientation of the tool.

Considering the influence of the tool gravity force on the force sensor output, the mathematical model of the force sensor output (Figure 3) can be defined as following by using the Euler angles:

(4) $$\left\{\begin{array}{c} F_{x}=-mg*\sin\left(\theta_{1}\right)*\cos\left(\theta_{2}+d\right)+a\\ F_{y}=-mg*\sin\left(\theta_{1}\right)*\sin\left(\theta_{2}+d\right)+b\\ F_{z}=mg*\cos\left(\theta_{1}\right)+c \end{array}\right.$$

where $F_{x}$, $F_{y}$ and $F_{z}$ are the outputs of the force sensor. The unknown parameters are the mass *m*, and the constant coefficients *a*, *b*, and *c*. *g* represents gravity which is 9.8 m/s^2^. The angles $\theta_{1}$ and $\theta_{2}$ are the orientation angles of the tool pose. *d* is the deviation error angle around the *z*-axis from the tool installation.

If there is no interaction force on the tool, the output of the force sensor represents the effects of the tool gravity, as follows:

(5) $$F_{S}=F_{ToolGravity}$$

With the acquired data including tool pose and the output of the force sensor without interaction force, the parameters listed in Equation (4) can be obtained with the CF technique. The detailed results of CF can be found in our previous works [30].

#### 3.1.2. Model-Free Tool Gravity Identification Using Mnn

However, it is difficult to identify an accurate mathematical model due to manufacturing and assembly variances. Moreover, there should be a deviation error on $\theta_{1}$ because the tool cannot be installed in a straight way. Hence it is difficult to project the gravity force of the tool on the force sensor with the model proposed in (4). The precise mapping relation between the gravity and the rotation angles of the tool direction is, therefore, too complex to model. Hence, we propose a novel model-free based method to map the relation between the gravity force and the rotation angles as follows:

(6) $$F_{ToolGravity}=f\left(\theta_{x},\theta_{y},\theta_{z}\right),$$

where *f* is a nonlinear unknown function and the corresponding Euler angles $\theta_{x},\theta_{y},\theta_{z}$ are the multiple inputs of the function.

In the past decades, the ANN approach became the most popular method for modeling linear and nonlinear regression problems [31]. Although the capability of ANN models extends to establishing any complex function and nonlinear relationship between a certain set of inputs and outputs with multiple dimensions [32], some limitations remain and need to be solved, such as over-fitting, under-fitting and time-consuming.

**Figure 4:**
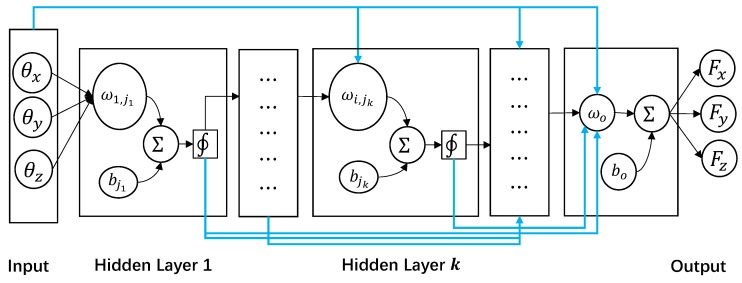
The architecture of muiltilayer neural networks (MNN) for mapping the 3D degree of angles $\left[\theta_{x},\theta_{y},\theta_{z}\right]$ and 3D forces $\left[F_{x},F_{y},F_{z}\right]$. The feed-forward (FF)-MNN model is shown by all of the black lines, while the cascade-forward (CF)-MNN model connects the outputs of each previous layers to the current layer (shown with blue lines).

To enhance the predictive performance of the built model, such as high-accuracy, stability and high-speed, this work adopts two types of MNN methods to establish a regression model, namely FF-MNN and CF-MNN. Figure 4 shows the structure of MNN mapping the 3D inputs (the degree of angles $\left[\theta_{x},\theta_{y},\theta_{z}\right]$) and 3D outputs (the forces $\left[F_{x},F_{y},F_{z}\right]$). In contrast to the FF-MNN model which connects all neurons in each hidden layer for fitting the nonlinear function, the CF-MNN [33,34] model can acquire the information from all of the previous layers by connecting the outputs to the latter networks (shown in the blue lines).

Hence, the MNN-based nonlinear model for mapping the time-varying multiple inputs $x_{t}={\left[\theta_{x},\theta_{y},\theta_{z}\right]}_{t}$ to the multi-outputs $y_{t}={\left[F_{x},F_{y},F_{z}\right]}_{t}$ can be defined as follows:

(7) $$y_{t}=f_{t}\left(x_{t},\Theta \right)=f_{t}\left(x_{t},\left\{\omega_{i,j_{k}},b_{j_{k}}\right\}\right),$$

where the whole parameter set Θ accounts for all of the nonlinear weights matrix $\omega_{i,j_{k}},i,j_{k}\in R^{+}$ and bias $b_{j_{k}}$ in each layer where *k* is the order of layer. By substituting the related parameters into FF-MNN model, the nonlinear function can be written as:

(8) $$y=b_{o}+w_{o}\sum_{k=1}^{K}\Phi_{k}\left(\sum_{i=1}^{N}\sum_{j=1}^{M}\omega_{i,j_{k}}\gamma_{t}^{j_{k}}+b_{j_{k}}\right).$$

$\Phi_{k}$ is the activation function. In this article, we chose the Broyden–Fletcher–Goldfarb–Shanno (BFGS) quasi-newton function [35]. $\omega_{o}$ and $b_{o}$ are the parameters of output layer. $\gamma^{j_{k}}$ and $b_{j_{k}}$ are the outputs of *j*th neuron. Similarly, the CF-MNN model can be expressed as follows:

(9) $$y=b_{o}+w_{o}\sum_{k=2}^{K}\Phi_{k}\left(\sum_{k=1}^{K-1}\Phi_{k}\left(\sum_{i=1}^{N}\sum_{j=1}^{M}\omega_{i,j_{k}}\gamma_{t}^{j_{k}}+b_{j_{k}}\right)\right).$$

It uses not only the output of the former layer but reserves also all of the previous information for obtaining the final results. The MNN regression model aims to search the optimal parameter set Θ by computing the minimum least squares between the predicted result $\hat{y}$ and the real value *y* as follows:

(10) $$\Theta =\underset{\Theta }{\mathrm{argmin}}\sum_{t=1}^{n}{\left({\hat{y}}_{t}-y_{t}\right)}^{2}=\underset{\Theta }{\mathrm{argmin}}{∥\hat{y}-y∥}_{2}^{2}.$$

There are three common evaluation indices to measure the performance of the built MNN models, namely mean square error (MSE), root mean square error (RMSE) and Pearson correlation coefficient ρ defined in Equation (11).

(11) $$\begin{array}{c} \mathrm{MSE}={\left[\sum_{t=1}^{N}{\left(\frac{\hat{y}-y}{t}\right)}^{2}\right]}_{\left(1\right)}\\ \mathrm{RMSE}={\sqrt{\left[\sum_{t=1}^{N}{\left(\frac{\hat{y}-y}{t}\right)}^{2}\right]}}_{\left(2\right)}\\ \rho =\frac{1}{N-1}\sum_{t=1}^{N}\left(\frac{{\hat{y}}_{t}-\mu_{\hat{y}}}{\sigma_{\hat{y}}}\right){\left(\frac{y_{t}-\mu_{y}}{\sigma_{y}}\right)}_{\left(3\right)}. \end{array}$$

The time *t* can be regarded as the number of observations. $\mu_{\hat{y}}$ and $\sigma_{\hat{y}}$ are the average and standard deviation of $\hat{y}$, while $\mu_{y}$ and $\sigma_{y}$ are the same values of *y*. The best score for the Pearson correlation coefficient ρ is 1 while for the other errors it is 0. The MNN model aims to predict the force close to the measured value.

### 3.2. Force Sensor Calibration

**Figure 5:**
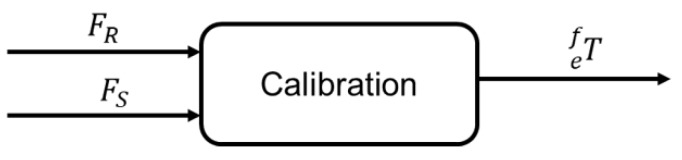
Force sensor calibration.

The force sensor is a particularly significant source of feedback in robotic applications to measure forces along *x*-, *y*- and *z*-axes at the end effector of the robot increasing sensitivity of the surgeon [36]. To achieve the best possible transparency, the force sensor should be calibrated in the system where it will be used. The SVD of a matrix is a linear algebra tool that has been successfully applied to a wide variety of domains [37]. In this work, the SVD method [38] is adopted to figure out the transformation (calibration) matrix ${}_{e}^{f}T$ between the reference frames of the slave’s end effector and the force sensor, as depicted in Figure 5. Figure 5 demonstrates the input-output of our calibration method, where $F_{R}\in R^{1\times 3}$, $F_{S}\in R^{1\times 3}$, are the robot and sensor forces, respectively. ${}_{e}^{f}T\in R^{4\times 4}$ is the calibration matrix.

Here, ${}_{e}^{f}T$ is comprised of a rotation component ${}_{e}^{f}R\in R^{3\times 3}$ and a translation component ${}_{e}^{f}t\in R^{1\times 3}$. The final transformation formula can be written as follows:

(12) $$F_{h}{=}_{e}^{f}R\left(F_{S}-F_{ToolGravity}\right){+}_{e}^{f}t,$$

where $F_{h}\in R^{1\times 3}$ is the final haptic force on the end effector of the robot frame.

**Figure 6:**
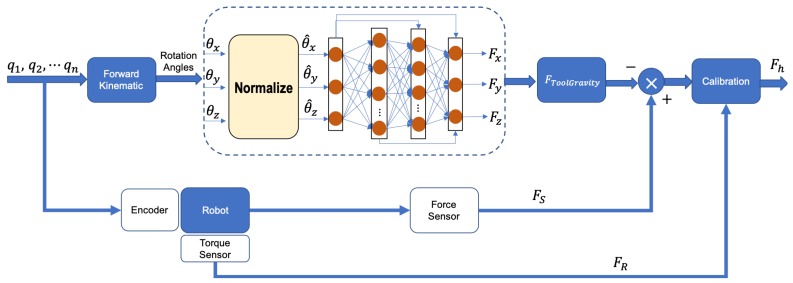
Overview of the identification and calibration procedure using MNN.

After the identification and calibration, the output of the system is in the end effector frame, which is able to achieve accurate force sensing for the robot control. The overview of the procedure of the tool dynamic identification and calibration using MNN is shown in Figure 6.

## 4. Experimental Validation and Results

### 4.1. System Description

**Figure 7:**
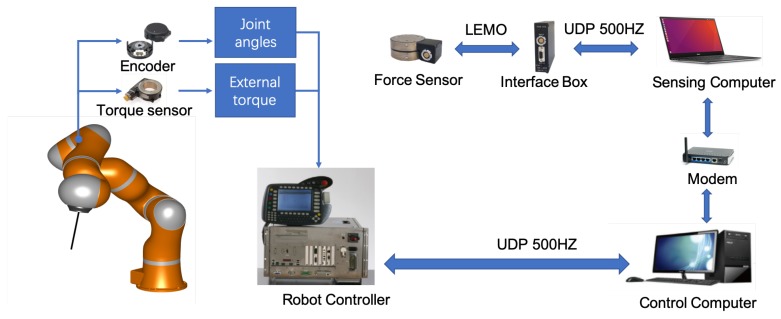
Overview of the developed robot control system. Multiple sensors including an encoder, a torque sensor and a force sensor, are adopted to collect the corresponding motion and force data.

**Figure 8:**
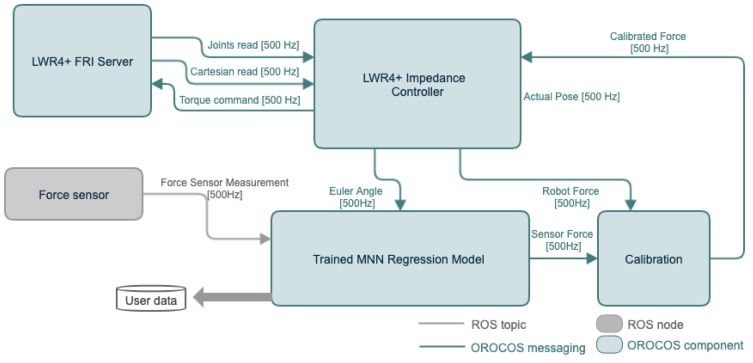
Overview of the developed software system. The “LWR4+ Impedance Controller” is adopted to allow hands-on control to move the surgical robot arm by hands. The “force sensor” is developed using Robot Operating System (ROS) and it communicate with the Open Robotic Control Software (OROCOS) by ROS topic with a frequency of 500 Hz.

A brief description of the robot system developed in this project is shown in Figure 7. A redundant robot (LWR4+, KUKA, Augsburg, Germany) served as the slave manipulator torque-controlled through fast research interface (FRI), which could provide direct low-level real-time access to the robot controller (KRC) at rates of up to 1 kHz [39]. The software system was developed with OROCOS (Open Robotic Control Software, http://www.orocos.org/) with a real-time Xenomai-patched Linux kernel and ROS (Robot Operating System, http://www.ros.org/) kinetic in Ubuntu [40]. To guarantee control frequency, force sensor, ROS node and OROCOS torque controller were executed on separate computers with UDP communication [41] between each other: the control loop and the sensing ROS node was executed on separate computers, as shown in Figure 8. The system consists of:

- a seven DoFs LightWeight robotic arm (LWR4+, KUKA, Augsburg, Germany) as slave device.
- a six-axis force sensor (M8128C6, SRI, Nanning, China) [42] that has the purpose of measuring interaction force between the surgical tool-tip and the environment.

### 4.2. Tool Dynamic Identification

Firstly, hands-on control was activated to allow the user to move the robot arm without touching the robot tool and the force sensor. In this way, two groups of data were collected for estimation and validation. All collected signals had 74,195 samples which are divided into a training dataset (42,148 samples) for building the regression models and a testing dataset (32,047 samples) for evaluating the performance of the built models.

#### 4.2.1. Model-Based Tool Dynamic Identification Using Cf

Firstly, we introduced CF for tool dynamic identification. As mentioned before, dynamic identification was implemented with respect to current end effector orientation. By utilizing the CF technique, the constant unknown parameter *m*, and the constant coefficients *a*, *b* and *c* can be obtained with the first group of sampled data (42,148 samples). Then, the obtained parameters were placed in the mathematical model to predict the force on the force sensor, which is expressed as follows:

(13) $$\left\{\begin{array}{c} F_{x,\mathrm{estimated}}=-0.3434*g*\sin\left(\theta_{1}\right)*\cos\left(\theta_{2}+1.401\right)+0.6\\ F_{y,\mathrm{estimated}}=-0.3434*g*\sin\left(\theta_{1}\right)*\sin\left(\theta_{2}+1.401\right)+1.1\\ F_{z,\mathrm{estimated}}=0.3434*g*\cos\left(\theta_{1}\right)+2.0\;\;\;\;\;\;\;\;\;\;\;\;\;\;\;\;\;\;\;\;\;\; \end{array}\right.$$

After the tool dynamic identification with CF, we validated the obtained mathematical model with the testing data (32,047 samples). The RMSE on the *x*-axis was calculated as 1.696, while it was 2.931 on the *y*-axis, and 1.057 was obtained on the *z*-axis. The overall RMSE of the testing data of the prediction error of the norm of the force is 5.684.

#### 4.2.2. Model-Free Tool Dynamic Identification Using Mnn

The model-based tool identification method has shown a big error due to the inaccurate mathematical model. To solve this problem, we adopted MNN to model the tool dynamic without using a mathematical model. To implement a nonlinear regression model for enhanced accuracy, fast computation and strong stability, the experiments were designed to compare and discuss the performance among eight single and multiple layer NN models with different numbers of nodes. As shown in Table 2, the first four MNN models (M1-M4) have two hidden layers with different numbers of nodes. For example, M1 had 30 and 15 neurons in the first and second hidden layers, respectively. Hence, we adopted the notation [30,15] to represent the nodes. The models M1 and M2 use CF networks, while M3 and M4 adopt FF networks. To enhance the validity of the comparison results, four single hidden layer NN models (M5-M8) based on CF and FF networks, namely CF-single layer neural network (SNN) and FF-SNN, are chosen in the experiments.

The four aspects for the evaluation the performance of the eight models comprise the training time, the online predictive time, the regression errors (i.e., MSE and RMSE), and the correlation coefficient ρ. To avoid the phenomenon of over-fitting and under-fitting and to find the most stable NN, each of the models was tested 30 times.

**Figure 9:**
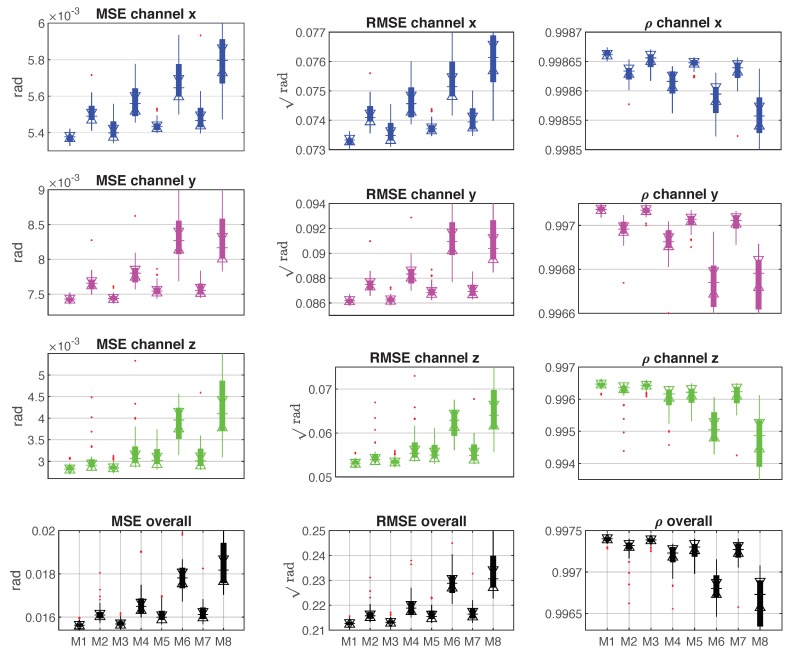
The comparison results of mean square error (MSE), root mean square error (RMSE) and correlation coefficient ρ among models M1–M8 on the training dataset.

The first experiment was designed for proving the reconstruction ability of the adopted models. Figure 9 shows the results of comparing MSE, RMSE and correlation coefficient ρ among models M1 to M8 on the training dataset. The top three rows show the MSE, RMSE and ρ of each channel (*x*, *y* and *z*). The fourth row displays the sum of errors (MSE and RMSE) and the average of coefficients ρ. By observing the eight pictures on the left two columns, M1 and M3 obtain the lowest errors for modeling the tool dynamic, while M1 had a lower error on the data collected from channel x than model M3. In addition, M1 was the most robust model due to its smaller standard deviation in respect to the other models. Similarly, by comparing the results of correlation coefficient ρ (in the third column), M1 proved to be the best reconstruction model for mapping the Euler angles to the force on the training dataset.

The errors and coefficient only illustrate the ability of reconstruction accuracy and model stability. However, the time-consuming of training a NN model is an other significant issue which is needed to discuss. Table 3 compares the total training times among M1 to M8. Although the M1 model obtains the best performance for predicting the training dataset, it costs 53.33 s (average) to build the model while M3 needs only 46.19 s. Observing the results of training time, the SNN models cost less time than the MNN models, and smaller numbers of nodes can save training time.

**Figure 10:**
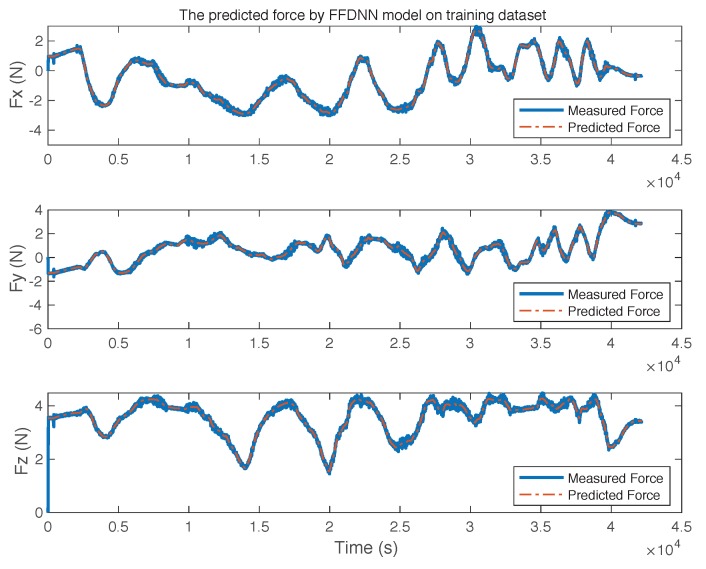
The predicted force of M1 model on the training dataset.

Figure 10 shows the measured and predicted force (by the M1 model) of the three channels. By observing the trend and difference between the measured curves and the predicted curves, the M1 model almost completely reconstructed the shape of the original curves.

**Figure 11:**
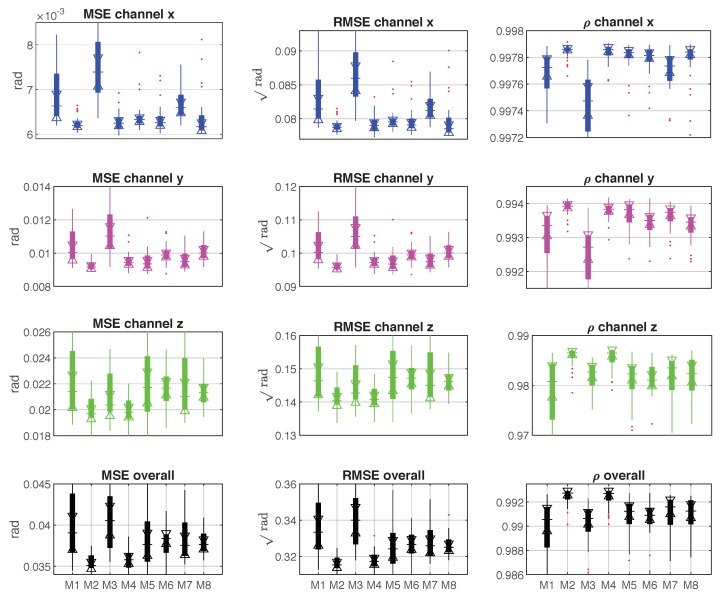
The comparison results of MSE, RMSE and correlation coefficient ρ among M1–M8 on the testing dataset.

After acquiring the regression models, their performance has to be validated on the new collected (testing) dataset. Similarly to the above, the errors (MSE and RMSE), correlation coefficient ρ and testing time were the important indices for measuring the eight models. Figure 11 shows the comparison results of MSE, RMSE and ρ among M1 to M8. Contrary to the results obtained on the training dataset, the M1 and M3 got the worst errors and ρ values with respect to the other models. On the other hand, M2 and M4 are proven to be the best models for predicting force on the testing dataset. Because both of them acquired lower errors and higher ρ values than the other models.

The M1 and M3 approach obtain the worst errors with respect to the other models, but the difference between M1, M3 and other models is not too much. For example, the overall MSE of M1 model is about 0.015, while the best results computed by M8 is 0.018. The M1 and M3 had overfitting problems. The M2 and M4 models acquired better performance on the testing dataset because they are fit the outputs on that dataset. However, when they were adopted on the training dataset, they were underfitting. Finally, the M1 and M3 models were not suitable for predicting on the testing dataset.

The average testing time was a significant index to evaluate detection speed of the built model. Table 4 displays the average and standard deviation of the testing time. Although the M2 model can acquire the highest regression accuracy, it spends 0.0230 s to predict a result which is slower than the other methods. However, this predictive speed was sufficient for the real experiment.

**Figure 12:**
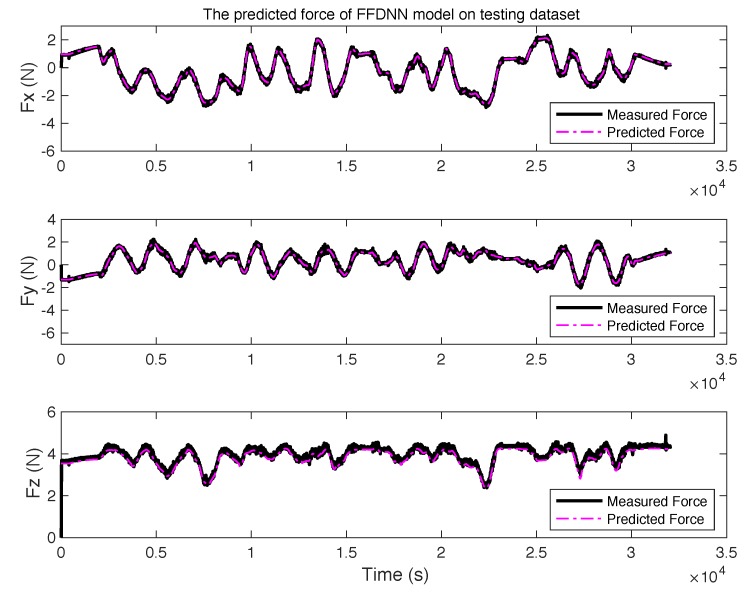
The predicted force of M2 model on the testing dataset.

Figure 12 shows the force predicted by the M2 model for each channel. By contrasting the difference between the measured and predicted force, the M2 model can track the real force.

### 4.3. Force Sensor Calibration

**Figure 13:**
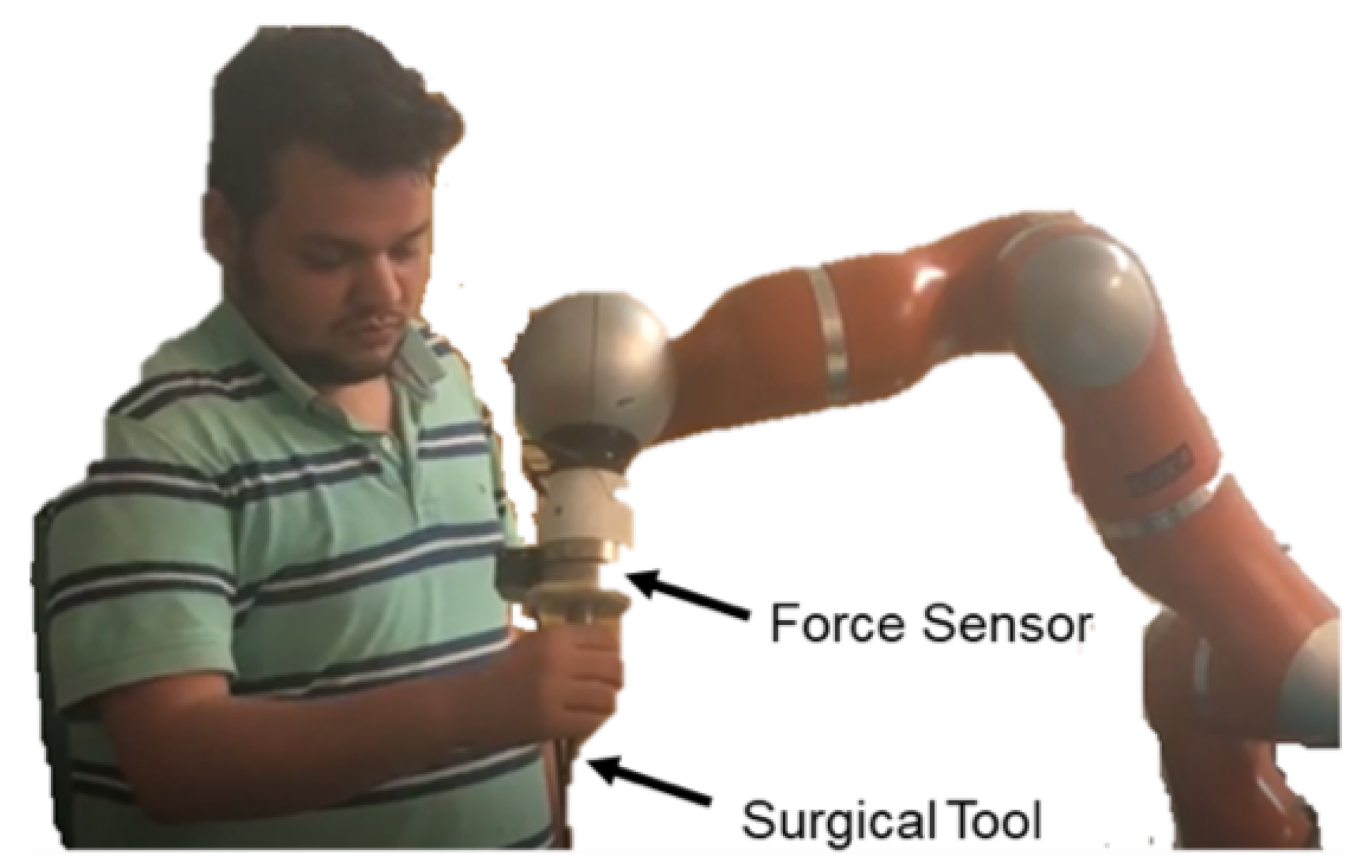
Hand-force applied on the tool of the robot.

**Figure 14:**
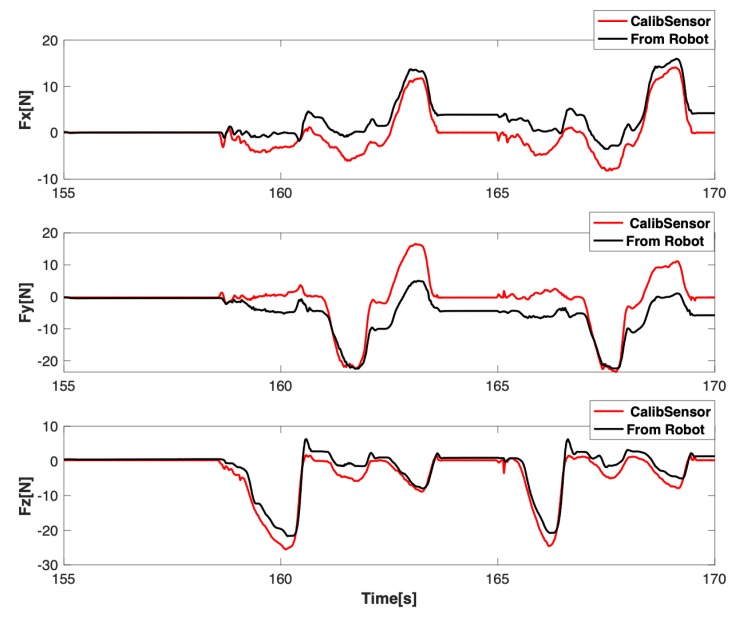
The calibrated force using singular value decomposition (SVD) after tool dynamic identification.

Although the interaction force is accurate after tool dynamic identification, the robot does not know the placement of the force sensor, especially for the orientation on its *z* axis. In this way, the *x* and *y* axes were not aligned with the robot coordination frame. Hence a calibration procedure was required to transfer the interaction force into the robot coordinates. As mentioned above, after identifying tool dynamic component by MNN, we performed the force sensor calibration by collecting more data using hands to touch the force sensor and then applying the SVD to solve the transformation matrix. It should be noticed that the influences from the gravity of the force sensor have been eliminated with the tool gravity identification using neural networks together. For the force on the robot end-effector, we used the software package provided by the KUKA Fri interface [40], and we assumed that there were no disturbances from the robot dynamic effects on it. As it is shown in Figure 13, the user applied hand force only on the tool. In this way, the force sensor and the robot were under the same external force. We collect the force from robot [39] and the force from the force sensor. To facilitate the availability of the force from the force sensor for the robot, the transformation matrix between them was calculated using an SVD based method. The results of the calibration are depicted in Figure 14.

## 5. Conclusions and Future Work

This paper presents a novel model-free based tool dynamic identification using MNN and force sensor calibration for accurate force sensing. Firstly, tool gravity force was identified by CF and MNN methods. The results showed that a model-free based tool dynamic identification using MNN is more accurate than a model-based identification using CF. Afterwards, force sensor calibration was implemented using SVD. Results showed that the calibrated force was able to represent the force from physical interaction.

Furthermore, the first CF-MNN model (M1) is proven as the best method for reconstructing the training dataset, while the first FF-MNN model (M2) is the best one to predict force on the testing dataset. It is observed that MNN approximation is more accurate than CF in the estimation of the gravity force, thus enhancing the accuracy of the tool dynamic identification and calibration for force sensing. It has been well known that Deep Neural Networks, such as convolutional neural networks (CNNs) and recurrent neural networks (RNN) [43], are capable of learning and modeling complex system [44] with high efficiency. Hence, further works will try to introduce DNN to model the gravity forces and compare their performances with the proposed methodology in this paper. The number of nodes is chosen through trial-and-error in this paper. We put the further analysis of how to select the neurons number as future works. An additional force sensor will also be placed to measure and validate the interaction force after processing. Future works will consider the robot dynamic effects on the force of its end-effector and achieve higher accuracy for the calibration procedure. Future work will also consider more challenging problems (e.g., dead-zone and time-delay) in our robot control framework. The system stability [45,46,47] and tracking accuracy [48,49] might not be guaranteed under these situations, which are a precondition for safety in robot control.

## Figures and Tables

**Table 1:** Denavit–Hartenberg (D-H) parameters of KUKA LWR4+ robot.

Link	$a_{i}$	$\alpha_{i}$	$d_{i}$	$\theta_{i}$
1	0	π/2	0	$q_{1}$
2	0	−π/2	0	$q_{2}$
3	0	−π/2	*L*	$q_{3}$
4	0	π/2	0	$q_{4}$
5	0	π/2	*M*	$q_{5}$
6	0	−π/2	0	$q_{6}$
7	0	0	0	$q_{7}$

**Table 2:** The selected models.

Label	M1	M2	M3	M4	M5	M6	M7	M8
model	CF-MNN	CF-MNN	FF-MNN	FF-MNN	CF-SNN	CF-SNN	FF-SNN	FF-SNN
nodes	[30,15]	[9,6]	[30,15]	[9,6]	[30]	[9]	[30]	[9]

**Table 3:** The comparison of training time among the eight models (M1–M8) in Table 2.

**Model**	**CF-MNN**	**CF-MNN**	**FF-MNN**	**FF-MNN**
Time (s)	53.33 ± 10.66	26.52 ± 5.71	46.19 ± 15.67	20.78 ± 4.31
**Model**	**CF-SNN**	**CF-SNN**	**FF-SNN**	**FF-SNN**
Time (s)	23.62 ± 5.44	17.06 ± 5.37	25.41 ± 6.93	19.49 ± 7.37

**Table 4:** The comparison of testing time among the eight models in Table 2 (M1–M8).

**Model**	**CF-MNN**	**CF-MNN**	**FF-MNN**	**FF-MNN**
Time (s)	0.0271 ± 0.0053	0.0230 ± 0.0017	0.0178 ± 0.0010	0.0158 ± 0.0015
**Model**	**CF-SNN**	**CF-SNN**	**FF-SNN**	**FF-SNN**
Time (s)	0.0182 ± 0.0013	0.0172 ± 0.0010	0.0138 ± 0.0017	0.0130 ± 0.0010

## Abbreviations

The following abbreviations are used in this manuscript:

MNN	Multi-layer neural network
CF	Curve fitting
FF-MNN	Feed-forward multi-layer neural network
CF-MNN	Cascade-forward Multi-layer neural network
FF-SNN	Feed-forward single layer neural network
CF-SNN	Cascade-forward single layer neural network
RA-MIS	Robot-assisted minimally invasive durgery
MIMO	Multi inputs multi outputs
SVD	Singular value decomposition
DoFs	Degrees of freedom
D-H	Denavit–Hartenberg
ROS	Robot Operating System
OROCOS	Open Robotic Control Software
